# Hybrid Modeling Method for a DEP Based Particle Manipulation

**DOI:** 10.3390/s130201730

**Published:** 2013-01-30

**Authors:** Mohamed Amine Miled, Antoine Gagne, Mohamad Sawan

**Affiliations:** Polytechnique Montreal, 2900 Edouard-Montpetit, Montreal, QC H3T 1J4, Canada; E-Mails: antoine.gagne-turcotte@polymtl.ca (A.G.); mohamad.sawan@polymtl.ca (M.S.)

**Keywords:** hybrid modeling, microfluidics, BioMEMS, particle manipulation

## Abstract

In this paper, a new modeling approach for Dielectrophoresis (DEP) based particle manipulation is presented. The proposed method fulfills missing links in finite element modeling between the multiphysic simulation and the biological behavior. This technique is amongst the first steps to develop a more complex platform covering several types of manipulations such as magnetophoresis and optics. The modeling approach is based on a hybrid interface using both ANSYS and MATLAB to link the propagation of the electrical field in the micro-channel to the particle motion. ANSYS is used to simulate the electrical propagation while MATLAB interprets the results to calculate cell displacement and send the new information to ANSYS for another turn. The beta version of the proposed technique takes into account particle shape, weight and its electrical properties. First obtained results are coherent with experimental results.

## 1. Introduction

Microelectromechanical systems (MEMS) already have certain maturity as a technology. They emerged in the 1970s, and they are now widespread amongst a variety of products and used in many home and entertainment applications. Current smartphones and videogame controllers are two excellent examples of such products. They are also present in most recent cars and printers. Because of their size, ease to manufacture, low cost and low power consumption, MEMS revolutionized many aspects of consumer electronics.

More recently, a new branch of MEMS emerged: BioMicroelectromechanical systems (BioMEMS) [1–3]. These devices are more oriented towards medical and biomedical applications, such as disease screening, DNA sequencing and separation and biological sample analysis. Because of their size and low cost, they can be used in patient monitoring for everyday life, such as in glucose-meters for diabetic patients, as they eradicate the necessity of costly and space-consuming medical equipment [4–6].

Currently, BioMEMS are not yet as mature as MEMS, and much development is still undergoing. However, the BioMEMS market is huge and promising such that a lot of effort is done to produce effective and low-cost designs. Based on the Yole report, MEMS market forecast and its various applications are expected to increase from 10,199 M$ in 2011 to 21,148 M$ in 2017 [7]. In addition, BioMEMS market is expected to grow by 18.6%, 24.6% and 32.5% in pharmaceutical and biological research, *in vitro* diagnostics and medical devices, respectively. Moreover, important markets are geographically localized in USA and Europe with 40% and 33% of world share respectively. This market is estimated to be 176.33 B$. Consequently, developing an efficient modeling tool and approach for BioMEMS is critical as it reduces time to market and stimulate research activities.

On the other hand, time to market is considered an important constraint for BioMEMS. Manufacturing such devices can take several months. Consequently, it is much more efficient to model the device before starting the manufacturing process in order to reduce the fabrication failure [8–11]. Simulating a device prior to manufacturing means to design it virtually and to simulate its behavior under certain circumstances. For example, the presence of electrodes in a microfluidic channel will generate a certain potential distribution when a voltage is applied. The simulation goal is to allow the researcher to know this potential distribution without actually manufacturing the device.

To model BioMEMS, many approaches have been explored [12–17]. First, it would be possible to simply use finite element modeling (FEM) to completely discretize a given BioMEMS geometry, then use complex non-linear differential equations to iteratively characterize the time-dependent behavior. Unfortunately, this method would require a lot of computations and possibly with unstable results, leading to erroneous data. As such, many simplifications can be made in order to simplify the problem and make it manageable and stable.

A first step to analyze a BioMEMS consists of considering the architecture without moving particles and using a time-independent simulation. A presented method by Voldman is used to determine the particle trapping efficiency of BioMEMS, which computes the dielectrophoretic, gravitational and hydrodynamic forces using mostly analytical results and finds the points where the force was zero to locate particles [18]. Another method proposed by Phillips consists of removing the use of volumetric discretization by using a boundary-element method [19]. Normally, in a moving-particle situation, the fluid volume should be remeshed when particles move, but by using boundary-elements, only the boundaries have to be remeshed, allowing much higher computational efficiency [20]. Many other methods present advantages and disadvantages towards simulations of multiphasic present in BioMEMS. It is possible to use finite-element models to characterize the reduced-order problem [21]. Another option is using a Precorrected-FFT method to analyze the electrostatic distribution [19]. Also a Multilevel Newton Method [22] or Full Lagrangian Schemes [23] have been explored.

However, commercial solutions such as ABAQUS, ANSYS and COMSOL provide convenient end-user functionalities to model and simulate virtually any geometry, with one or more physics domain, using finite element modeling. Since finite-element modeling is used, a lot of computations are necessary and the processing time is often larger than in the approaches previously introduced. Still, since it is more convenient, many researchers use this method to predict the behavior of their MEMS/BioMEMS devices.

We describe in this paper a new modeling approach based on FEM technique to model particle motion in a flowing liquid with an applied electrical field for DEP applications. Several published papers present simulation related to DEP effect [24–27]. However, their proposed approaches are limited to specific electrode architectures or electrical field propagation conditions and/or fluidic conditions, which are the standard approaches to study particle behaviors within DEP. Unfortunately, these techniques assume that medium and particle conductivity are homogeneous and the propagation of the electrical field is not affected by particle charge or shape. This is true when the electrical field is strong enough, but in the case of this work, a low-voltage DEP is used and consequently the electrical field is not propagated through all micro-channel depth. Consequently, in case of low voltage DEP electrode architecture, particle shape and charge are critical, and it is impossible to simulate the electrical field with conventional tools such as MATLAB, ANSYS or COMSOL, as they do not consider the biological aspect in electrical field modeling.

Consequently in this work, we demonstrate the first complete modeling approach of DEP-based particle motion in a micro-channel considering particle size and charge, electrode architecture, medium and particle conductivity and permittivity and liquid flow conditions. The proposed technique is not limited to any electrode architecture, particle size or fluidic conditions. In fact, ANSYS is used to build micro-channel and electrode geometry. MATLAB is used to insert particle in the ANSYS model and then ANSYS applies electric and fluidic loads. Unlike other techniques that propose a simulation of DEP parameters, such as electrical field or fluidic forces and then DEP forces are deduced, our modeling approach takes into consideration particle properties in the electrical field propagation and liquid flow to calculate DEP forces and apply it to calculate particle motion.

In this paper, a simplified modeling environment in MATLAB and linked to ANSYS is presented. All physics domains are considered uncoupled. The calculated force resulting from each iteration for each particle is used to determine its position during the next iteration in order to track particle displacement during simulation.

In the next section, we present relevant background on dielectrophoresis and BioMEMS Modeling. In Section 3, a theoretical description of the proposed model is detailed. Then in Section 4, the implementation procedure is introduced, and Section 5 shows obtained results and presents a comparison with experimental data.

## 2. DEP-Based BioMEMS Modeling Theory

### 2.1. Dielectrophoresis Background

**Figure 1.**
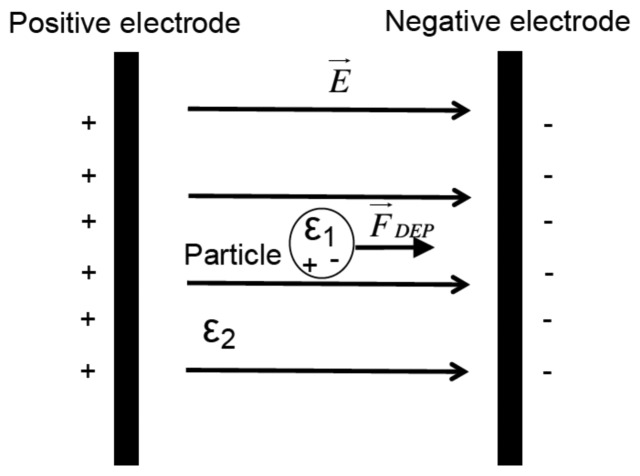
Dielectrophoretic effect using a particle with a permittivity of *ε*_1_ and medium permettivity *ε*_2_.

Dielectrophoresis is an electrical phenomenon that controls neutral-charge particle motion in a fluid induced by an inhomogeneous electrical field as shown in Figure 1.

The dielectrophorectic forces *F⃗_DEP_* governing particle motion are defined by Equation (1).

(1) $$<{\vec{F}}_{\text{DEP}}>=2\pi {ɛ}_{1}a^{3}\text{Re}\left[\underline{K}\left(\omega \right)\right]\nabla \left|\vec{E}\right|$$

where *Re*[.] refers to the real part of the complex number *K̲*(*ω*) and electrical field *E⃗* is defined by

(2) $$\vec{E}=-\nabla \phi_{\text{global}}$$

where *a*, *ε*_1_ and *ϕ_global_* are the radius of the particle, particle permittivity and global potential distribution in the micro-channel, respectively *ω* is equal to 2*πf* where *f* is the frequency of the electric field.

**Figure 2.**
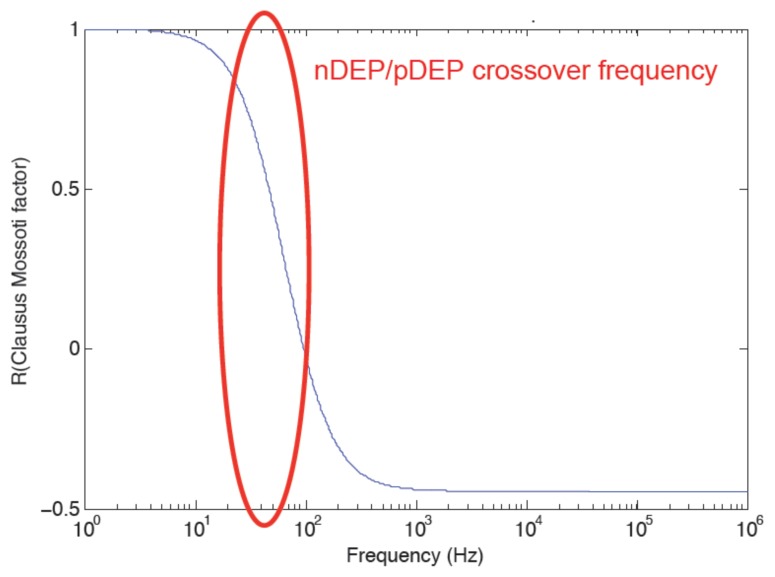
Variation of real part of the Claussius–Mossotti factor versus frequency showing the crossover frequency effect.

The Claussius–Mossotti factor *K̲*(*ω*) defines the frequency range of positive DEP and negative DEP, which correspond to attractive or repulsive effect, respectively The latter frequency range is defined by the crossover frequency as shown in Figure 2. *K̲*(*ω*) is given by Equation (3).

(3) $$\underline{K}\left(\omega \right)=\frac{{ɛ}_{2}-{ɛ}_{1}-j\left(\sigma_{2}-\sigma_{1}\right)/\omega }{{ɛ}_{2}+2{ɛ}_{1}-j\left(\sigma_{2}+2\sigma_{1}\right)/\omega }$$

where *ε*_2_ refers to medium permittivity and *σ*_1_ and *σ*_2_ are particles and medium conductivities, respectively The Claussius–Mossotti factor refers to the particle polarization. It is related to the phase lag that results from the dipoles formation in dielectric particles in a medium with an electric field.

### 2.2. Modeling Background

Modeling BioMEMS requires characterization of the electric, mechanical and hydro-dynamical behavior of the analyzed geometry with initial loads. The proposed way to simulate BioMEMS is separated in three different models: the electric model, the fluidic model and particles.

The main goal of this simulation is to track particle displacement based on its charge, weight and applied electrical field. The liquid flow and the electric field propagation monitor particles motion in micro-channel. Thus, the fluid and electric field contribution to particle displacement are detailed as follows.

First, the full hydrodynamic model requires solving the Navier–Stokes equation for the fluid that includes an electric field component, as shown in Equation (4), which is a complex nonlinear equation.

(4) $$\rho \frac{\delta \vec{v}}{\delta t}+\rho \left(\vec{v}\cdot \vec{\nabla }\right)=\rho \vec{g}+\rho_{e}\vec{E}-\vec{\nabla }p+\eta \nabla^{2}\vec{v}$$

where *p*, *ρ*, *v⃗*, *g⃗*, *η* are pressure distribution in liquid, liquid density, liquid velocity, gravity vector and viscosity, respectively. If particles are present in micro-channel, their displacement must be considered. Consequently, no steady state or harmonic behavior is considered to simplify the analysis. The electric model, in addition to contributing to the fluidic solution, is important for its electrophoretic and dielectrophoretic contribution and then on forces applied on particles. The electrophoretic force *F⃗_EP_* is computed from Equation (5) [28–32].

(5) $${\vec{F}}_{\text{EP}}=-q_{p}\vec{E}$$

where *q_p_* is the particle electric charge and *E⃗* the electric field.

Particles with their polarization also contribute to the resulting electric field. The potential induced from the dipoles is given by the approximation of a point-source dipole, as shown in Equation (6).

(6) $$\phi_{{\text{dipoles}}_{x,y,z}}=\frac{\rho_{\text{eff}}\cdot \text{cos}\theta }{4\pi {ɛ}_{2}a^{2}}$$

where *x*,*y*,*z* refer to particle coordinates, *θ* is the angle between the dipole direction and a point in the micro-channel, *ε*_2_ is the medium permittivity and *a* is the particle radius. *ρ_eff_* is the point-dipole and it is given by Equation (7).

(7) $$\rho_{\text{eff}}=4\pi {ɛ}_{1}\underline{K}a^{3}\vec{E}$$

The Claussius–Mossotti factor introduces a phase lag *φ* in *E⃗* in the point-dipole equation, which is computed from Equation (8).

(8) $$\varphi =\text{arct}g\left(\frac{\text{Im}\underline{\left(K\left(w\right)\right)}}{\text{Re}\underline{\left(K\left(w\right)\right)}}\right)$$

where $\text{Im}\underline{\left(K\left(w\right)\right)}$ and $\text{Re}\underline{\left(K\left(w\right)\right)}$ are the imaginary and real parts of the Claussius–Mossotti factor *K*(*w*).

If a particle has a fixed charge *q_p_* , then the electrical potential *ϕ_part_x,y,z__* at the position (x,y,z) is given by Equation (9).

(9) $$\phi_{{\text{part}}_{x,y,z}}=\sum \frac{{\left(q_{p}\right)}_{i}}{4\pi {ɛ}_{1}r}$$

Computing the electric component of the resulting force on particles, present in the BioMEMS, is straightforward using Equation (9). But the hydrodynamic force *F⃗_Hydrodynamic_* requires the computation of the pressure *P* over the surface *S* of each particle based on Equation (10).

(10) $${\vec{F}}_{\text{Hydrodynamic}}=\oint \text{PdS}$$

Theoretically, the hydrodynamic, electrophoretic and dielectrophoretic forces previously introduced make it possible to find the position of the particle after a given time-step, since the main goal of the simulation is to predict the position of the particle in a time-dependent simulation.

## 3. Proposed Modeling Method

The theory upon which the model is developed allows finding different parameters affecting particle displacement in a micro-channel. It can also be simplified as to quicken computations while giving an accurate prediction. This section presents the different assumptions, simplifications and details related to the implementation method.

The first assumption is that in the fluidic solution, particles can be dissociated from the observed geometry. This means that the computations in the fluidic regimen are done without taking into account particles. The main advantage of this assumption is the fact that solving the Navier–Stokes equations in finite-elements requires a meshing of the geometry, which is a computationally intensive operation.

Another problem associated with the meshing is that it can become unstable because of the stretching of the elements induced by the movement of particles. Since particles are moving in the fluid, the mesh must be recomputed for each iteration. If particles are removed from the simulation and the loads applied on the fluid are the same, the fluidic solution can be considered time-independent. If the electric contribution of the electric field to the fluid movement is considered negligible, the Navier–Stokes equation can then be computed only once. The result is considered steady state, if the flow is in laminar regimen.

Next, the different electric forces can be computed from the potential distribution on the electrodes. Equation (11) shows the electric field distribution from the potential distribution.

(11) $$-\nabla \cdot \left(\left[ɛ\right]\nabla V\right)+\frac{j}{\omega }\nabla \cdot \left(\left[\sigma \right]\nabla V\right)=0$$

where *ε* and *σ* are the permittivity and conductivity of the environment respectively. *V* is the applied voltage and *ω* is equal to 2*πf* where f is the frequency of applied voltages.

The dipoles induced from the presence of particles can be computed using Equation (11). Once the electric field distribution is known, the dielectrophoretic force is calculated using Equation (1). However, the dielectrophoretic force requires using the gradient of the electric field. Since this value is not needed for anything else, the gradient is simply computed at the current position of each particle. Equation (12) is an estimate of the x gradient at position x, y and z.

(12) $$\frac{\delta^{2}\phi }{\delta x^{2}}=\frac{\phi_{x+\Delta x,y,z}-2\phi_{x,y,z}+\phi_{x-\Delta x,y,z}}{\Delta x^{2}}$$

where Δ refers to the particle radius in the given direction and *ϕ* is the electrical potential of particle at different positions.

The next parameter needed to compute the dielectrophoretic force is the Claussius–Mossotti factor based on Equation (3).

Using these equations, the different forces applied on the particle can be computed using also Equations (1) and (2), though further simplification is possible. By solving the differential equations for the velocity versus the fluid velocity, for a given force value, the final velocity of a particle in a fluid can be calculated based on Equation (13).

(13) $$v={\text{ae}}^{-\text{bt}}+c$$

where *b* = −*k*/*m*, with *k* being the drag force and *m* the mass of particle. Assuming spherical human cell, *m* = 1*e* − 12 kg and *k* = 6*πμr* where *r* is the particle radius. Considering *μ* = 9*e* − 4, the water viscosity at 25 °*C*, *r* = 10*e* − 6m and *k* = 1.6965*e* − 007. Consequently, *b* is equal to 1.7e5. Thus, the exponential term fades rapidly, so that the particle reaches its final velocity *c* rapidly. *c* is the ratio of the total exerted force over the drag force. Based on experimental data, it has been shown that the force applied from a moving fluid on a spherical particle is depicted from Equation (14).

(14) $${\vec{F}}_{\text{fluid}}=6\pi \mu a\left(v-v_{0}\right)$$

where 6*πμa* is a constant defined by the fluid viscosity, *v* is the fluid velocity and *v*_0_ is particle velocity. Because of the fluid velocity component, the other forces do not contribute to an infinite velocity component, as they are linearly opposed to the fluid velocity. The electrophoretic velocity component is then computed based on Equation (15).

(15) $$v_{\text{electro}}=\frac{{\vec{F}}_{\text{EP}}}{6\pi \mu a}$$

The dielectrophoretic component is computed using Equation (16).

(16) $$v_{\text{dielectro}}=\frac{{\vec{F}}_{\text{DEP}}}{6\pi \mu a}$$

Based on Equation (14), computing the hydrodynamic component of the particle becomes trivial. Assuming that only forces induced by moving fluid are considered, the particle follows the fluid and falls according to gravity. If electrophoretic and dielectrophoretic forces are present, Equation (1) prevails.

## 4. Implementation of the Proposed Modeling Technique

Through ANSYS, it is possible to model a wide range of mechanical structures such as micro-channels and electrodes. It can also model electrical or magnetic field propagation, in addition to liquid flow. But it is not designed to model biological behavior of particles and cells. However COMSOL with particle tracing toolbox and ANSYS with fluent toolbox offer an interesting way to observe particle behavior, but they assume that particles have a steady state and do not consider the variation in particle electrical properties.

**Figure 3.**
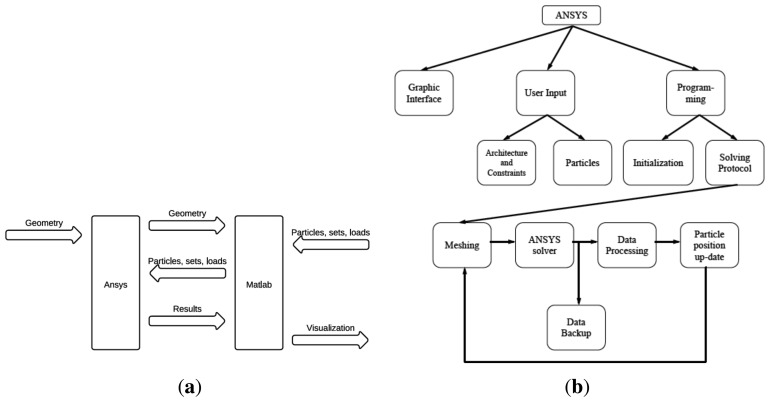
(**a**) ANSYS/MATLAB global and (**b**) detailed modeling of a particle motion.

**Figure 4.**
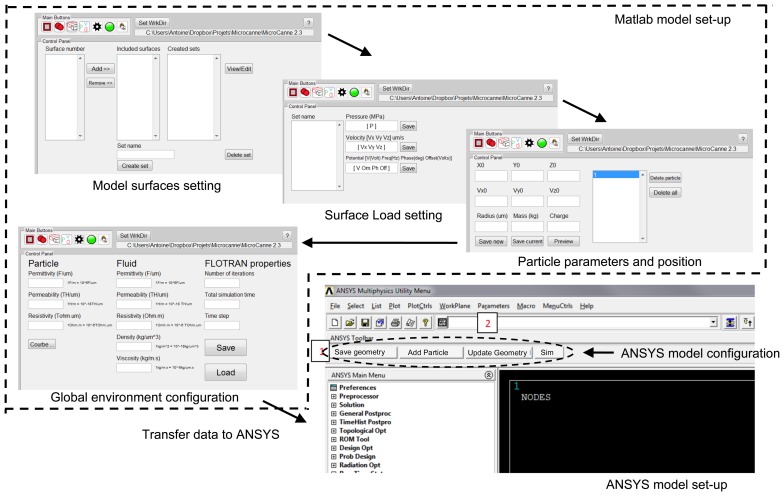
Modeling approach set-up steps using implemented MATLAB and ANSYS algorithms.

Consequently, the finite-element modeling software ANSYS is used, in conjunction with MATLAB as shown in Figure 3. The first step is the geometry production through ANSYSs geometry building functions. Thus, geometries are created in ANSYS and then exported to MATLAB. This format contains the list of all points, lines and areas used to produce the geometry. Using a custom graphical user interface, particle models are defined through MATLAB at the desired position with specified parameters such as mass, charge and radius as shown in Figure 4.

**Figure 5.**
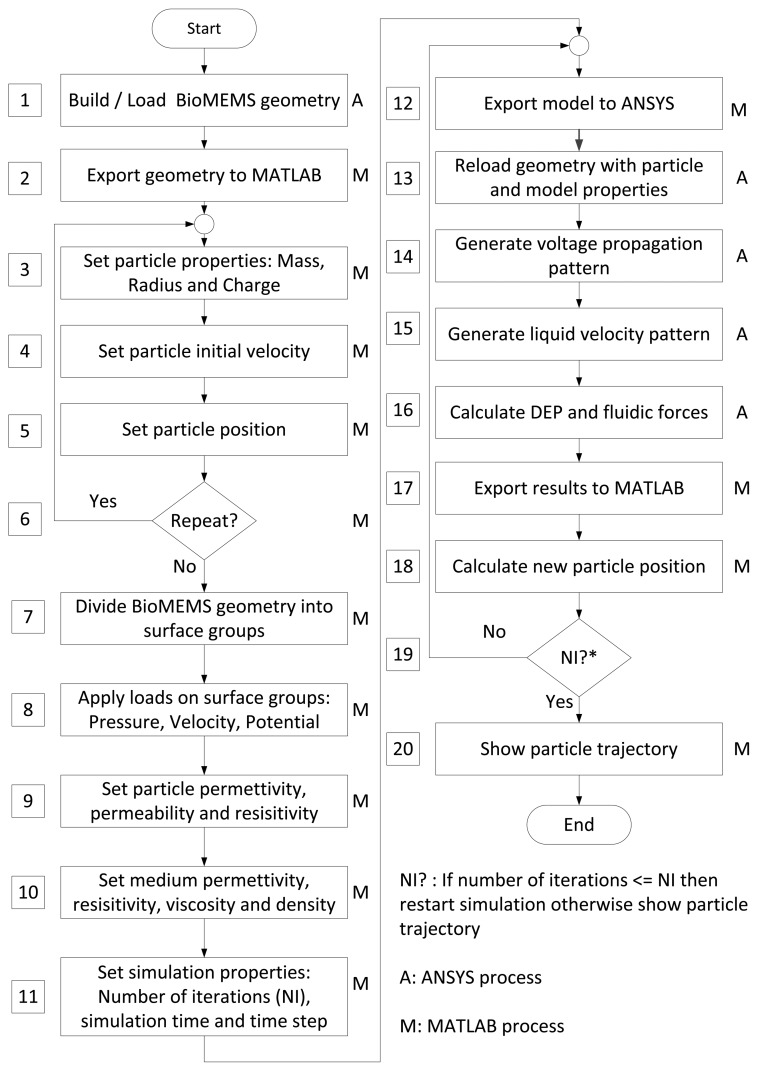
Detailed algorithm of proposed modeling approach for DEP based BioMEMS.

Load sets are then created within MATLAB interface. Different surfaces on which load will be applied are selected and grouped together. This is used to apply the same load to all surfaces contained in any electrode subset, or to apply a zero-velocity constraint on the micro-channel walls, as shown in Figures 4 and 5.

After setting different loads, the latter can be applied to each set of surfaces using MATLAB interface as shown in Figure 5. Pressure, velocity and electric loads are applied on each set of the geometry. These loads are saved in separate files and are used by both MATLAB and ANSYS. For each file, a list of the areas upon loads are applied is created. Then, the FLOTRAN's analysis properties are set along with the simulation parameters.

At this stage, global model analysis is started. First, MATLAB interface launches a while loop until data are received from ANSYS. The analysis is also started in ANSYS. After each iteration, the results are transferred to MATLAB. The simulation steps are as follows. First, the fluidic solution is generated. The fluidic solution is generated only once and it is reused throughout the simulation. To solve this part of the problem, ANSYS fetches the different loads written for pressure and velocity on the areas and applies them to the corresponding areas in the geometry. The solution is then generated using the default ANSYS solver for FLOTRAN elements. A while loop is then triggered, where ANSYS solves the electric simulation. Electrical field propagation results and in-channel pressure distribution are used in Equations (14–16) to compute the different velocity contributions of the hydrodynamic and electric forces. These contributions are sent to MATLAB, which computes and stores the incremented particle position. Upon termination, the data is saved and ready for analysis. Example of implementation of these algorithms are presented in Algorithm 1 and 2.


**Algorithm 1:** Implementation example of particle detection algorithm on ANSYS.

*del,*VOLT_LOAD**del,SizeAreas*DIM,SizeAreas,Array,1,1*VREAD,SizeAreas(1,1),SizeAreas,txt,,JIK,1,1 (1F14.10)*DIM,*VOLT*_*LOAD*,ARRAY,SizeAreas(1,1),5 !!!*GET,ParX,PARM,*VOLT*_*LOAD*,DIM,X*GET,ParY,PARM,*VOLT*_*LOAD*,DIM,Y*VREAD,*VOLT*_*LOAD*(1,1),*VOLT*_*LOAD*,txt,,JIK,ParY,ParX (5F41.20)!!!


**Figure 6.**
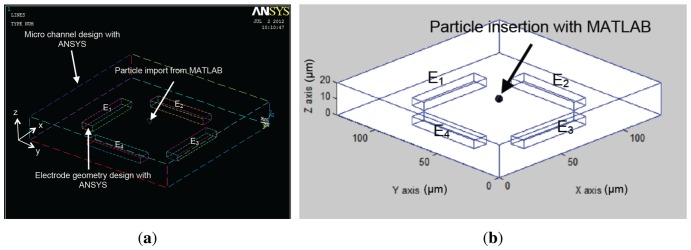
(**a**) Electrode modeling using ANSYS and (**b**) particle insertion with MATLAB in the micro-channel within a 4-electrode architecture.

**Figure 7.**
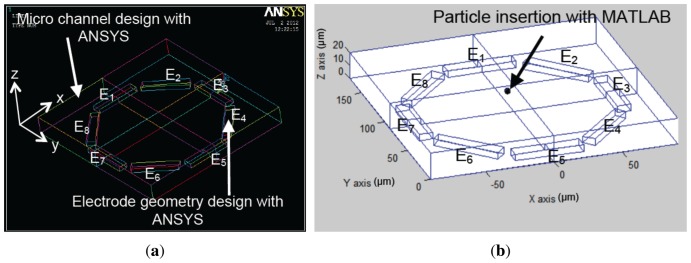
(**a**) ANSYS electrode modeling and (**b**) MATLAB particle insertion in the micro-channel within an 8-electrode architecture.

**Figure 8.**
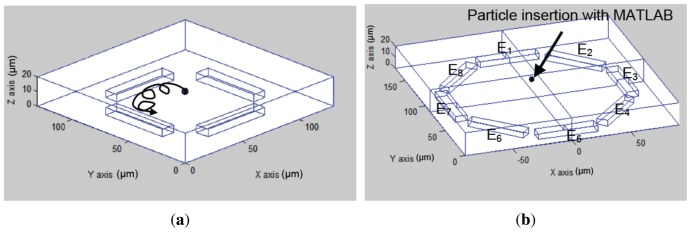
Particle motion approximation (**a**) based on Figure 9(a) with a 4-electrode architecture and (**b**) based on Figure 10(a) with 8-electrode architecture.

**Figure 9.**
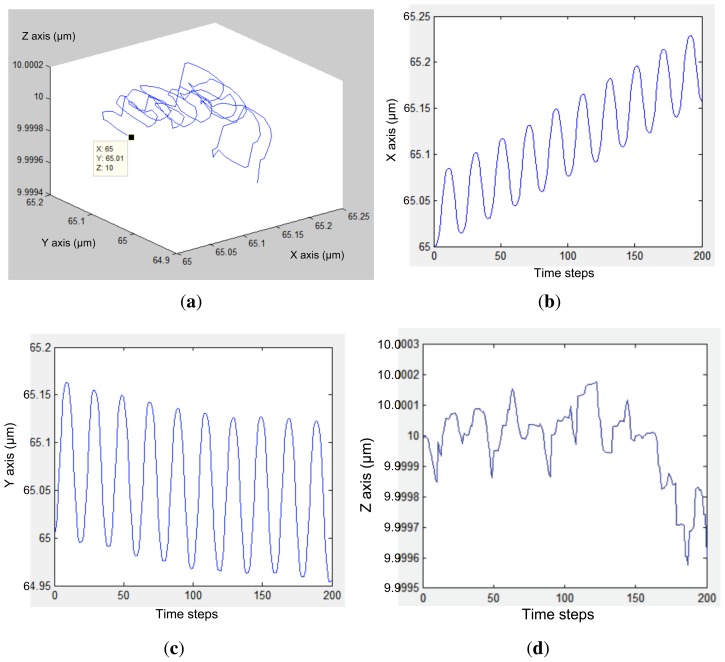
(**a**) Particle 3D-displacement in a 4-electrode architecture, where (**b**–**d**) show X, Y and Z displacement respectively. X, Y and Z axis are defined in Figure 6(a).

**Figure 10.**
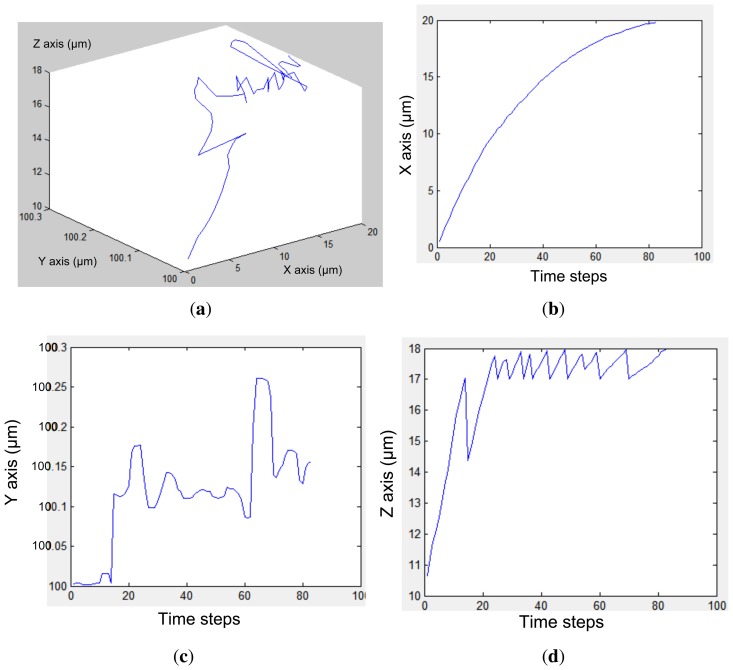
(**a**) Particle 3D-displacement in an 8-electrode architecture, where (**b**–**d**) show X, Y and Z displacements respectively. X, Y and Z axis are defined in Figure 7(a).

Figures 6 and 7 present the electrode models used in this work. Figure 8(a,b) show an approximation of particle trajectory based on Figures 9(a) and 10(a), respectively. It can be seen from these figures that particle has a helical trajectory that cannot be observed experimentally, the diameter of the helical trajectory is 150 nm and particles are repulsed to the top of the channel. The latter results are critical as it can be used as a method for z-separation of particles. Indeed Figures 9(d) and 10(d) both show that particle are suspended at 18 *μm* and 10 *μm* depth respectively. These data cannot be obtained from experimental results based on Figure 16 as they are 2D images. Figures 9(d) and 10(d) are related to two different electrode architectures. It can be seen that the 8-electrode architecture is not optimized for z-separation in this case as particle are pushed until the top of channel, unlike the 4-electrode architecture where particles are suspended in the middle of the channel depth (10 *μm*). The main objective of the following modeling approach is to provide information for a most efficient particle separation technique based not only on electrical field or particle or medium properties, but also on electrode architecture.


**Algorithm 2:** Implementation example of data acquisition algorithm from ANSYS to MATLAB.
**if**
*A* == *0*
**then** **for**
*i* = *1:size(LINES1,1)*
**do**  KP1=LINES1(i);  KP2=LINES2(i);  **if**
*KP1* = *0*
**then**   POSX = [KPS1(KP1) KPS1(KP2)];   POSY = [KPS2(KP1) KPS2(KP2)];   POSZ = [KPS3(KP1) KPS3(KP2)];   line(POSX,POSY,POSZ);  **else**   line(POSX,POSY,POSZ);  **end** **end****else** **for**
*k* = *1:size(A,1)*
**do**  *h*_*lines* = [AREAS1(A(k)) AREAS2(A(k)) …;*h*_*lines* = *h*_*lines*(*h*_*lines* = 0); **end** **for**
*i* = *1:size(LINES1,1)*
**do**  **if**
*(find(h*_*lines*==*i))*
**then**   KP1=LINES1(i);;   KP2=LINES2(i);;   **if**
*KP1* =*0*
**then**    POSX = [KPS1(KP1) KPS1(KP2)];;    POSY = [KPS2(KP1) KPS2(KP2)];;    POSZ = [KPS3(KP1) KPS3(KP2)];;    line(POSX,POSY,POSZ,’color’,’r’,’linewidth’,3);   **else**    line(POSX,POSY,POSZ,’color’,’r’,’linewidth’,3);   **end**  **else**   KP1=LINES1(i);;   KP2=LINES2(i);;   **for**
*KP1* =*0*
**do**    POSX = [KPS1(KP1) KPS1(KP2)];;    POSY = [KPS2(KP1) KPS2(KP2)];;    POSZ = [KPS3(KP1) KPS3(KP2)];;    line(POSX,POSY,POSZ);;   **end**  **end** **end** KP1=LINES1(i);**end**


## 5. Simulation and Experimental Results

### 5.1. Simulation Results

**Figure 11.**
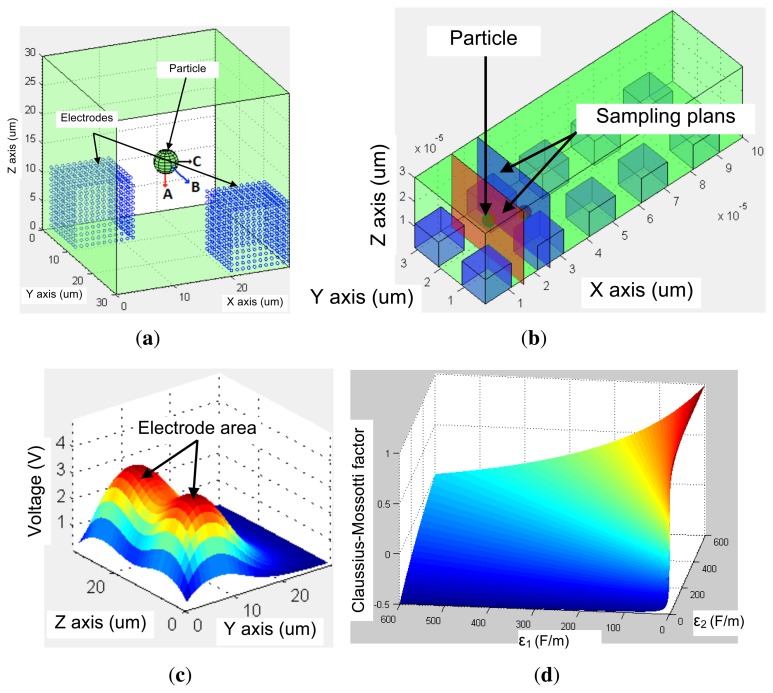
(**a**) The red arrow (**A**) represents the dielectrophoretic force, the blue arrow (**B**) represents the fluid force and the black arrow (**C**) represents the electrophoretic force, (**b**) Sampling procedure of the proposed model, (**c**) Voltage propagation and (**d**) Claussius–Mossotti factor calculation.

Figure 11 shows the proposed model behavior to achieve the simulations. First the model, sample the channel into different section depending on required steps as shown in Figure 11(b). Then, the voltage propagation imported from ANSYS and presented in Figure 11(c) is used to calculate electrical field propagation based on Equation (2). The latter is used to calculate the electrophoretic forces *F⃗_EP_*. The model also calculates the Claussius–Mossoti factor to find the dielectrophoretic forces *F⃗_DEP_* as shown in Figure 11(b). All calculated forces, in addition to the fluidic forces *F⃗_fluid_*, are applied on particle as shown in Figure 11(a).

Simulations have been performed to compare two different geometries. A fluid initial velocity was imposed to the microfluidic channels. Sine wave voltage loads are applied on the different electrodes with different phase shift. Tests have been carried out in the same conditions as in a real BioMEMS in order to compare the simulated and experimental results. The first experiment has been carried out in a 4-electrode architecture, where a phase offset of *π*/2 was applied for each electrode regarding the previous one as shown in Figure 6.

**Figure 12.**
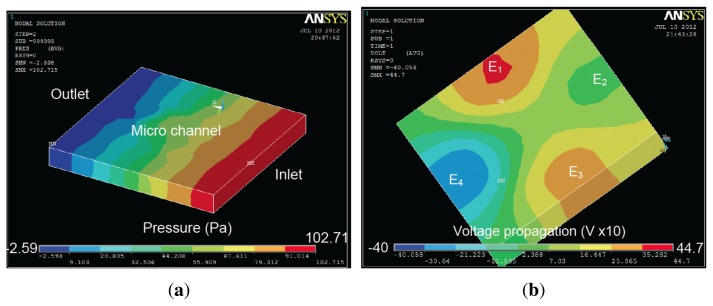
ANSYS results in the case of a 4-electrode architecture: (**a**) pressure and (**b**) voltage distribution in the micro-channel. Voltage distribution is multiplied by 10 in ANSYS due to simulation constraints.

The proposed modeling method provides accurate information regarding particle displacement in the micro-channel, as presented in Figures 9 and 10 at a frequency of 1 MHz and an applied voltage amplitude of 5 V. These results are obtained based on Voltage and Pressure distribution from ANSYS, as shown in Figure 12. The latter is then transferred to MATLAB in order to compile results and update particle position before sending back the new position to ANSYS.

**Figure 13.**
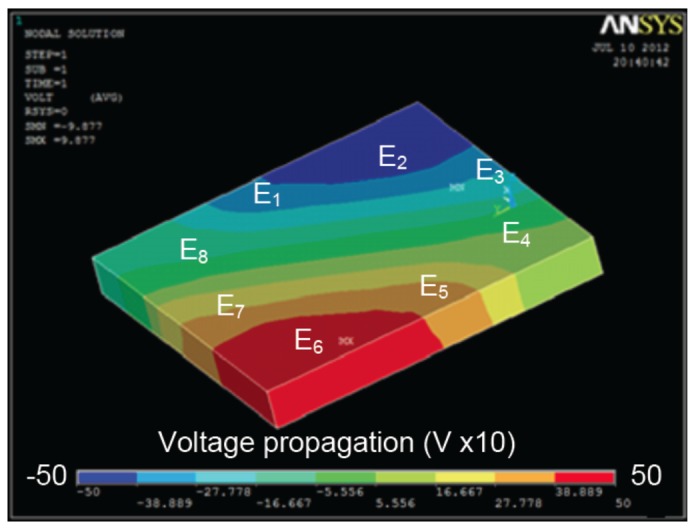
Voltage distribution in the case of 8-electrode architecture using ANSYS. Voltage distribution is multiplied by 10 in ANSYS due to simulation constraints.

The second experiment has been carried out on an 8-electrode array in an octagonal pattern. The electrodes are offset by *π*/4 regarding the previous one. The geometry is shown in Figure 7 and ANSYS results are presented in Figure 13.

**Figure 14.**
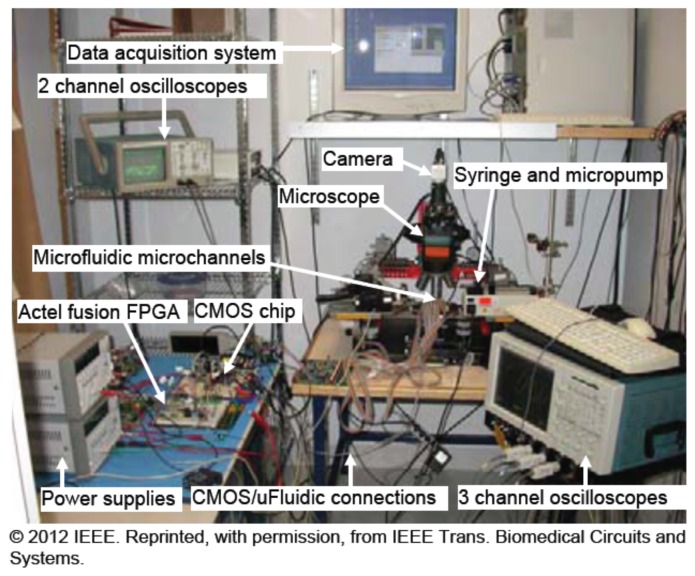
System set-up to test DEP effect on microspheres injected in a custom design of electrode architecture [33].

Following the presented simulation results, the main advantage of such method consists of giving more accurate results regarding particle displacement in addition to electrical field propagation [34]. Figure 14 shows the system set-up to test the BioMEMS.

### 5.2. Experimental Set-Up

**Figure 15.**
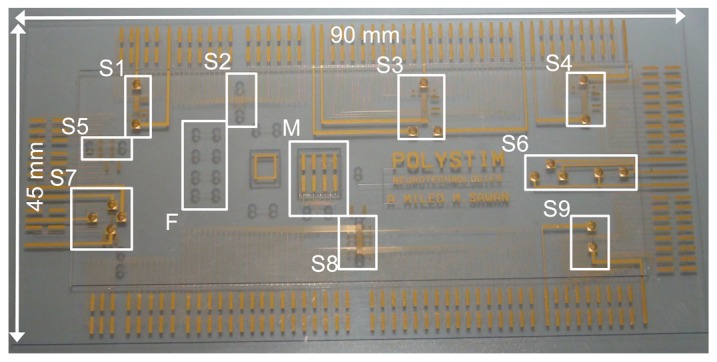
Microfluidic fabricated device using LioniX process.

The fabricated microfluidic substrate is made with two pieces of Borofloat glass, each with a thickness of 500 *μm*. The in-channel electrode thickness is 200 nm, the access holes diameter is 1.5 mm and the micro-channel depth is 40 *μm*. The microfluidic platform contains 9 different architectures, as shown in Figure 15. Only architectures S3 and S9 are used for experimental results. Other architectures are implemented for validation purpose only. Micro-tubes are sealed to access holes with epoxy Epotek-731 and connected to Cole-Parmer micropump.

Injected microspheres are 0.97 *μm* diameter polystyrene microspheres (dyed red) from Bangs Laboratories. 40 *μL* of microsphere solution is mixed with 1.8 mL deionized water to reduce particle concentration. 4 sine wave voltages are applied on electrodes with 0°, 90°, 180° and 270° phase respectively. The speed of flowing liquid is controlled by a micropump and is set to 100 nL/min. Sine wave voltages are generated by a FPGA Spartan3A from Xilinx and digital to analog converters.

### 5.3. Experimental Results

**Figure 16.**
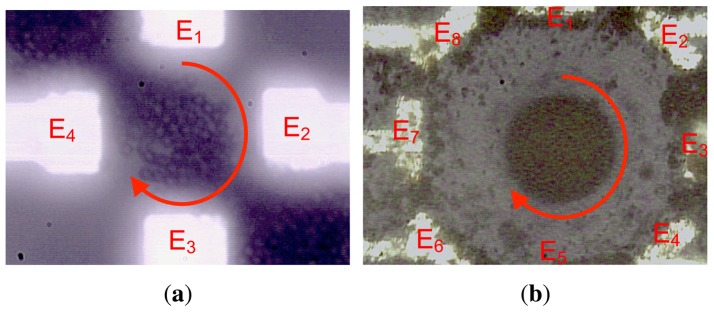
Experimental results of 0.97 *μm* diameter polystyrene microspheres (dyed red) from Bangs Laboratories exposed to DEP forces within (**a**) a 4-electrode architecture and (**b**) an 8-electrode architecture.

Figure 16 highlights main experimental results of the simulated model with the same number of electrodes (E); *i.e.*, 4 and 8 electrodes for the first and second presented architectures respectively [33,35]. The latter cannot provide the exact 3D position of particles in the micro-channel. However, Figures 9 and 10 show that particle displacement in the z-axis differs depending on the used electrode. In the case of Figure 9, particles are not steady in the z-axis, however in the case of 8-electrode architecture, particles are pushed toward the top side of the micro-channel.

Particle observation was made with Olympus BX51 microscope to track particles with QImaging software and shown in Figure 16. Particle tracking can be experimentally achieved if particles can be easily identified and their concentration is low. In the actual experiment, the concentration is very high for observation purpose. Also, all particles are exactly the same, so no software can track them as they cannot be distinguished individually—the actual acquisition speed of the camera is 5 frames per second, whereas the electrical field frequency is higher than 10 kHz. Consequently, the software cannot proceed with particle tracking with reliable results.

**Figure 17.**
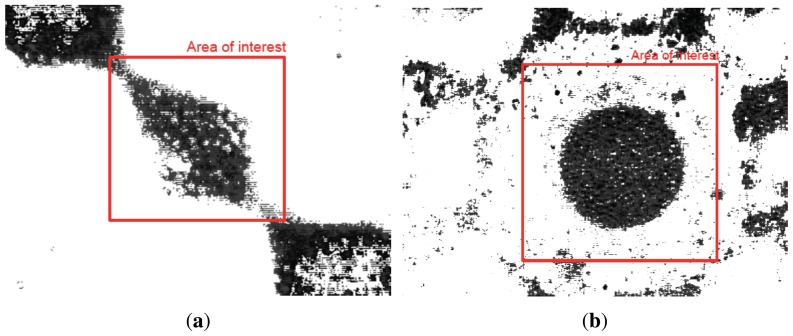
Image processing with MATLAB of captured image from Suss microscope using 0.97 *μm* diameter polystyrene microspheres (dyed red) from Bangs Laboratories exposed to DEP forces within (**a**) a 4-electrode architecture and (**b**) an 8-electrode architecture.

The objective of the actual modeling approach is to study the particle motion when an electrical field is applied within flowing liquid. Experimental results cannot provide enough data regarding particle motion except a limited 2D motion when the concentration of particles is low. In fact, when particle concentration is high with a cloudy dispersion, it is not possible to track particles even with an advanced algorithm because the software cannot identify the particles. Indeed, we tried to track particles with both QImaging software and an advanced developed algorithm on MATLAB, and both approaches failed to track particles as shown in Figure 17. Through MATLAB, the only useful information that can be acquired in this case is the particle number and concentration, *i.e.*, 61 particles with a concentration of 43.95% and 768 particles with a concentration of 63.7% in the case of Figure 17(a,b), respectively, in the area of interest.

Indeed, electrical field frequency is varying between 10 kHz and 1 MHz, which keeps particle tracking impossible with QImaging software. Applying lower frequency signal for observation purpose (under 5 kHz) leads to electro osmosis that generates air bubble, and consequently electrode oxidation is observed and DEP forces are stopped.

**Figure 18.**
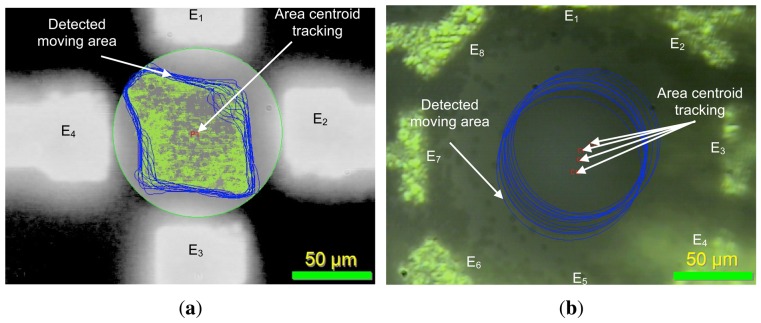
2D experimental tracking in the case of (**a**) 4 and (**b**) 8 electrode architectures.

To validate the model results experimentally, we used the recently released ImagePro Premier software from MediaCybernetics. The software can do a 2D tracking of particles by making an average of global motion of a selected area by area centroid tracking as shown in Figure 18. This approach is coherent with this paper's particle motion example in the way that it starts from the center of each electrode architecture and is limited to 2D tracking only. Thus the comparison between the experiment and the model is limited to the X and Y axes only.

**Figure 19.**
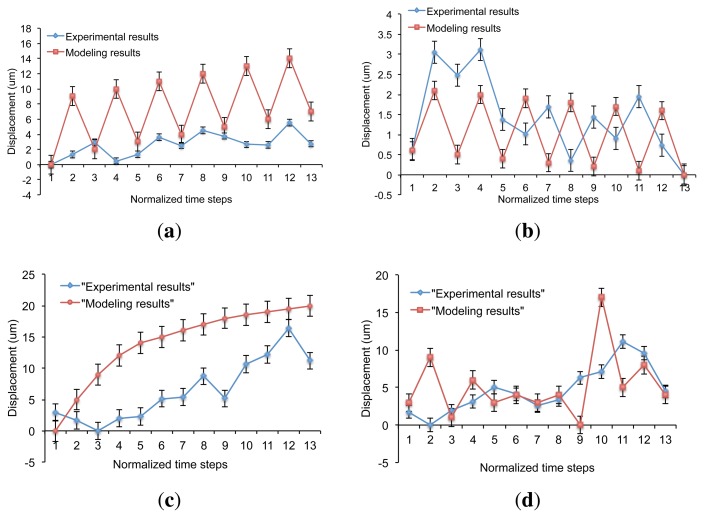
2D experimental result comparison for (**a**) X and (**b**) Y axes in the case of 4-electrode architecture and (**c**) X and (**d**) Y axes in the case of 8-electrode architecture.

The graphs in Figure 19 show the variation of X and Y displacement of particles in the case of 4-and 8-electrode architectures. It can be seen that in both cases, the modeling and experimental curves are close to each other. In addition, we observe a similar motion pattern for all curves in major cases. However, it is important to note that modeling results are related directly to the particle's motion, while the experimental ones are an approximation of the particle's motion based on the motion of area centroid.

In the case of DEP manipulation, based on simulation results provided in Figures 9 and 10, particle separation can be processed in z-axis in addition to x and y axes, which helps to design 3D electrode structure. By using the proposed modeling method, it is easier to predict particle behavior before BioMEMS fabrication.

## 6. Discussion

The main advantage of the proposed model compared with other softwares consists of its high versatility. Indeed, unlike ANSYS and COMSOL, the proposed model takes into consideration not only the architecture of the microfluidic structure, but also applied voltages on electrodes, type of signals (AC or DC), and particle properties, especially the charge of the particles. In the latter case, ANSYS and COMSOL are limited to ions and electrons to consider the charge of particles. This is mainly due to meshing problem in all FEM software, as Navier–Stokes equations in finite-elements require an intensive calculation, which can lead to non-convergence problem. With our proposed method, particles are not moving when the meshing is done. The obtained results from ANSYS are exported to MATLAB, and then forces are applied on particles based on calculation done by ANSYS to calculate particle motion. Following this step, a remeshing is used for another step.

ANSYS was selected due to the high programming flexibility offered by the software. However, we are currently studying another solution to test the model with COMSOL to compare obtained results with ANSYS and to consider wall effects.

Tables 1 and 2 summarize the features of the proposed model regarding major FEM softwares such as ANSYS and COMSOL.

In the present work, we are particularly interested in BioMEMS for particle manipulation and analysis in real-time. In fact, real-time monitoring of neurotransmitters is of great importance for understanding the chemical behavior of the brain. Neurotransmitters are very small molecules and have different electrical and physical properties. DEP is well established for particles that are a few micrometers in size. Nevertheless, whether DEP can still be functional with nanoparticles such as neurotransmitters remains to be proven. Thus, an advanced modeling of DEP with a fully configurable environment is mandatory to understand neurotransmitter's behavior in an implantable device.

More precisely, our purpose is to elaborate a new modeling approach to study biological particle motion when exposed to external electrical field through in-channel electrodes. Up to now, it is not possible with any commercial software to model or simulate such behavior because particle properties are changing over the time. For example, when an electrical field is applied, biological cells exchange ions (Na^+^, Cl^2−^, K^+^) with medium. This ion exchange changes medium conductivity and affects DEP forces. Furthermore, the objective is to implement a separation method based on frequency identification of neurotransmitters. Also, COMSOL and ANSYS are not initially designed to study the biological behavior.

Finally, results presented in this paper correspond to a steady charge and conductivity of both medium and particles. However, it is easy to add a non-steady state medium and particle, as these parameters are set in MATLAB and can be redefined for each simulation step. Furthermore, the proposed model is limited to DEP manipulation, though it can be extended to other manipulations if they are implemented in MATLAB. The concept of this model consists of using ANSYS as a platform to get results from applied loads for different external fields, such as electrical or magnetic fields. MATLAB used these results in combination with particle properties to calculate particle displacement in a loop process (MATLAB-ANSYS). Ultimately, this proposed approach can be integrated directly to FEM software.

## 7. Conclusions

This work highlights an important issue in the BioMEMS field, *i.e.*, the Bio-multiphysics simulation. It is not trivial to find a commercial tool that can handle both the engineering and the biological aspects of a device by taking into account the fabrication and electrical features in addition to the biological behavior. The proposed method is based on ANSYS as it offers advanced programming interface. However, the proposed approach requires a lot of CPU resources and is limited to the DEP manipulation. A further work is undertaken to enlarge it to the biological manipulation, as well as to add the magnetophoresis method and the corresponding sensing operations. As it is the first modeling approach, we are still working on several improvements on the biological aspects. The next step, which is undertaken, consists of considering the particle and its close medium as one entity.

## Figures and Tables

**Table 1.** Comparaison of different modeling techniques.

	**ANSYS**	**COMSOL**	**This work**
Multi-physics toolbox	Does not include biological aspect	Does not include biological aspect	Particle shape and charges considered
Simulation technique	Superposition	Simulation results can be linked by equations	Simulation results are dependent on each other
Particle tracking	Computational fluid dynamics (CFD) module	Particle tracing module	Does not need any additional module
Charged particle simulation	Charged particle limited to ions	Charged particle limited to ions and electrons	No limitation

**Table 2.** Main proposed model features.

**Feature**	**Used equation**	**Input parameter**
Particle charge	*F⃗_EP_* = −*q_P_E⃗*	*q_p_*
Particle shape	*<F⃗_DEP_*> = 2*πε*_1_*a*^3^*Re*[*K̲*(*ω*)] ∇|*E⃗*|	a
Particle permettivity	$\underline{K}\left(\omega \right)=\frac{{ɛ}_{2}-{ɛ}_{1}-j\left(\sigma_{2}-\sigma_{1}\right)/\omega }{{ɛ}_{2}+2{ɛ}_{1}-j\left(\sigma_{2}+2\sigma_{1}\right)/\omega }$	*ε*_1_, *σ*_1_
Medium permettivity	$\underline{K}\left(\omega \right)=\frac{{ɛ}_{2}-{ɛ}_{1}-j\left(\sigma_{2}-\sigma_{1}\right)/\omega }{{ɛ}_{2}+2{ɛ}_{1}-j\left(\sigma_{2}+2\sigma_{1}\right)/\omega }$	*ε*_2_, *σ*_2_
Fluid velocity	*F⃗_fluid_* = 6*πμa*(*v* − *v*_0_)	*v*
